# Comparison of chimeric mouse-human and humanized anti-CD25 monoclonal antibodies for steroid-refractory acute graft-versus-host disease

**DOI:** 10.3389/fimmu.2025.1660452

**Published:** 2026-01-02

**Authors:** Xinhui Zheng, Yunxia Zhou, Yawei Zheng, Ni Lu, Yigeng Cao, Weihua Zhai, Jialin Wei, Donglin Yang, Rongli Zhang, Aiming Pang, Sizhou Feng, Mingzhe Han, Erlie Jiang, Xin Chen

**Affiliations:** State Key Laboratory of Experimental Hematology, National Clinical Research Center for Blood Diseases, Haihe Laboratory of Cell Ecosystem, Institute of Hematology & Blood Diseases Hospital, Chinese Academy of Medical Sciences & Peking Union Medical College, Tianjin, China

**Keywords:** anti-CD25 monoclonal antibodies, basiliximab, graft-versus-host disease, stem cell transplantation, xenopax

## Abstract

**Introduction:**

Steroid-refractory acute graft-versus-host disease (SR-aGVHD) represents a severe and persistent complication that can arise following allogeneichematopoietic stem cell transplantation (allo-HSCT). This study aimed toassess the effectiveness and safety of basiliximab compared with those of a humanized anti-CD25 monoclonal antibody (xenopax) in the treatment of SR-aGVHD in patients who underwent allo-HSCT.

**Methods:**

This retrospective trial included 32 patients diagnosed with SR-aGVHD who were administered xenopax at 1 mg/kg on days 1, 4, and 8, and weekly thereafter until aGVHD severity was reduced to below grade 2. A historical cohort of 37 patients received basiliximab, which is a chimeric mouse-human anti-CD25 antibody.

**Results:**

The overall response (OR) rate on day 28 was not significantly different between The xenopax and basiliximab groups, with rates of 82% and 72%, respectively (p=0.57). Additionally, no differences were observed between the groups regarding the safety profile. The 1-year overall survival (OS) and non-relapse mortality (NRM) rates in the xenopax and basiliximab cohorts were 64% versus 40% (p=0.06) and 45% versus 48% (p=0.46), respectively.

**Discussion:**

In conclusion, no significant differences were observed in efficacy or adverse events between chimeric mouse-human and humanized anti-CD25 monoclonal antibodies for the treatment of SR-aGVHD. Further studies with larger cohorts are necessary to validate these findings.

## Introduction

Acute graft-versus-host disease (aGVHD) is a major complication that can occur after allogeneic hematopoietic stem cell transplantation (allo-HSCT), thereby contributing to substantial morbidity and mortality (1, 2). Corticosteroids are the primary treatment option for aGVHD, but the overall response rate is approximately 50%, and patients exhibiting steroid-resistant aGVHD (SR-aGVHD) often experience poor long-term outcomes (3). Although ruxolitinib is now the established standard of care (1), suboptimal response rates and heterogeneous clinical practices necessitate the exploration of additional therapeutic agents.

Anti-CD25 monoclonal antibodies, which specifically target interleukin-2 receptor alpha (IL-2Rα), play a crucial role in inhibiting T lymphocyte activation and have demonstrated efficacy in mitigating GVHD (4–6). The effectiveness and safety of these antibodies have been substantiated by numerous studies. Among them, basiliximab is the most widely utilized and has a high response rate (7, 8). However, the availability of basiliximab can be limited compared to domestic alternatives, highlighting the need to evaluate other therapeutic options (9).

Xenopax, a humanized anti-CD25 monoclonal antibody developed by 3SBIO (Shenyang, China), received approval from the China State Food and Drug Administration for the prevention of acute rejection after kidney transplantation. A previous clinical trial revealed that among 64 patients with SR-aGVHD receiving xenopax, the overall response rates were 48.4% (31/64), 53.1% (34/64), and 79.7% (51/64) on days 7, 14, and 28 post-treatment, respectively (10). While xenopax has demonstrated promising efficacy in treating SR-aGVHD, further clinical data are necessary to substantiate these findings. Additionally, it is essential to explore the factors influencing the efficacy of CD25 monoclonal antibody treatment for SR-aGVHD and to compare the efficacy and safety of xenopax with those of other commonly used anti-CD25 monoclonal antibodies.

Therefore, this single-center retrospective trial evaluated the efficacy and safety of xenopax among patients with SR-aGVHD. Additionally, the efficacy and safety of xenopax was compared with that of basiliximab, which was administered to a historical cohort of patients at the same center.

## Methods

### Study design and patients

From December 2021 to May 2023, this trial enrolled 32 patients with SR-aGVHD who underwent HSCT. The participants received xenopax at a dose of 1 mg/kg on days 1, 4, and 8, and weekly thereafter until the severity of aGVHD decreased to below grade 2. The exclusion criteria for the study were as follows: graft source including bone marrow, grade I aGVHD, overlap syndrome in chronic GVHD, aGVHD induced by cellular or immunotherapy, and incomplete patient data. For comparative analysis, a historical cohort comprising 37 consecutive SR-aGVHD patients who were administered basiliximab as a secondary treatment option was selected. The same exclusion criteria were applied to the historical cohort. Basiliximab was administered to these patients at a dose of 20 mg on days 1, 3 (or 4), and 8, and weekly thereafter until the severity of aGVHD was reduced to below grade 2.

The study protocol was approved by the Ethics Committee of Blood Diseases Hospital and the Institutional Review Board. All patients provided both written and oral consent.

### Definitions and endpoints

Acute GVHD was evaluated via the modified Glucksberg criteria (11), whereas chronic GVHD was defined in accordance with the National Institutes of Health (NIH) guidelines (12). In the isolated intestinal cohort, diagnosis was established based on clinical findings consistent with the Glucksberg criteria, following the clinical exclusion of other etiologies, particularly gastrointestinal infections. The definition of SR-aGVHD was established as follows: worsening of aGVHD within three days of initiating treatment with methylprednisolone at a dose of 2 mg/kg/day; absence of improvement within seven days of treatment; or treatment failure during the tapering phase of steroid administration. HSCT was performed as outlined in a previously published study (13).

The primary endpoint was the overall response rate (ORR) on day 28, encompassing both complete and partial response (CR and PR, respectively). The secondary endpoints included the ORR on day 56; overall survival (OS), defined as the interval from treatment initiation to the date of death or last follow-up; cumulative incidence of relapse (CIR), measured from treatment to relapse; non-relapse mortality (NRM), defined as the period from treatment until the date of death from any cause without prior relapse; and the incidence of chronic GVHD. Clinical risk stratification by Minnesota criteria have been previously reported (14). Safety assessments, with a particular focus on infections, were conducted in accordance with version 5.0 of the Common Terminology Criteria for Adverse Events (CTCAE).

### Statistical analysis

Descriptive statistics were utilized to encapsulate the demographic and transplant-specific data of the patient cohort. Continuous variables are represented as medians accompanied by interquartile ranges (IQRs) and were analyzed by either Student’s t tests or Mann–Whitney U tests, whereas categorical variables were assessed via chi-square tests or Fisher’s exact tests. Both univariate and multivariate analyses were conducted via logistic regression models. Overall survival (OS) was assessed via the Kaplan–Meier method, with p values determined via the log-rank test. Furthermore, we conducted a competing risk regression analysis utilizing the Fine and Gray model, wherein relapse and non-relapse mortality (NRM) were considered competing risks, as were relapse and death in the context of chronic graft-versus-host disease (cGVHD). To account for selection bias, we applied a propensity score–based standardized inverse probability of treatment weighting (sIPTW) method and subsequently reassessed all study outcomes. The propensity scores were estimated using a logistic regression model that included the following clinically relevant baseline covariates: use of ruxolitinib combination therapy, onset-to-treatment interval for aGVHD, administered doses, and aGVHD status prior to the initiation of anti-CD25 monoclonal antibody therapy. Statistical analyses were conducted with R software (v4.1.4), and two-tailed p values less than 0.05 indicated statistical significance.

## Results

### Patient characteristics

Between December 2021 and May 2023, this trial enrolled 32 patients (23 males and 9 females), receiving xenopax as second-line therapy for SR-aGVHD. In contrast, the historical cohort receiving basiliximab, which served as the control group, comprised 37 patients (26 males and 11 females), enrolled between January 2015 and December 2018. Compared with the control group, xenopax-treated patients had greater use of ruxolitinib combination therapy (p=0.086), shorter onset-to-treatment intervals for aGVHD (p=0.045), and received more doses (p=0.002). Other baseline characteristics were comparable between the two groups. After the application of SIPTW, the covariates were relatively balanced across the study groups (**Table 1**).

**Table 1:** Comparison of the demographic and transplant-specific profiles of the patients in the xenopax and basiliximab cohorts.

Characteristics	Before IPTW		P	After IPTW		P
	Basiliximab (n=37)	Xenopax (n=32)		Basiliximab (n=30.1)	Xenopax (n=28.8)	
Median age, years (IQR)	40.00 [29.00, 47.00]	48.50 [34.75, 51.25]	0.116	40.42 [29.00, 46.58]	37.36 [28.66, 49.00]	0.922
Patient gender, n (%)						
Female	11 (29.7)	9 (28.1)	1	6.6 (21.9)	12.5 (43.3)	0.123
Male	26 (70.3)	23 (71.9)		23.5 (78.1)	16.3 (56.7)	
Diagnosis, n (%)						
AL	18 (48.6)	18 (56.2)	0.766	16.2 (53.7)	17.8 (61.9)	0.817
MDS	11 (29.7)	9 (28.1)		8.3 (27.4)	7.1 (24.5)	
other*	8 (21.6)	5 (15.6)		5.7 (18.8)	3.9 (13.6)	
Disease status before HSCT, n (%)						
CR1	16 (43.2)	17 (53.1)	0.556	14.3 (47.6)	12.6 (43.6)	0.944
≥CR2	15 (40.5)	9 (28.1)		10.9 (36.3)	10.6 (36.8)	
no chemotherapy	6 (16.2)	6 (18.8)		4.9 (16.1)	5.6 (19.6)	
HCT- CI, n (%)						
0	26 (70.3)	21 (65.6)	0.878	21.4 (70.9)	17.1 (59.5)	0.45
≥1	11 (29.7)	11 (34.4)		8.8 (29.1)	11.7 (40.5)	
Transplantation type (%)						
HID	19 (51.4)	20 (62.5)	0.325	15.8 (52.5)	17.4 (60.3)	0.489
MSD	16 (43.2)	12 (37.5)		13.0 (43.1)	11.4 (39.7)	
URD	2 (5.4)	0 (0.0)		1.3 (4.4)	0.0 (0.0)	
Donor/recipient gender match, n (%)						
Female to male	8 (21.6)	8 (25.0)	0.964	7.4 (24.5)	7.4 (25.8)	0.919
Others	29 (78.4)	24 (75.0)		22.8 (75.5)	21.3 (74.2)	
ABO match, n (%)						
Match	16 (43.2)	20 (62.5)	0.175	13.4 (44.5)	20.2 (70.0)	0.079
Mismatch	21 (56.8)	12 (37.5)		16.7 (55.5)	8.6 (30.0)	
Conditioning regimen, n (%)						
BU- based	31 (83.8)	27 (84.4)	1	25.3 (83.8)	23.1 (80.4)	0.765
TBI- based	6 (16.2)	5 (15.6)		4.9 (16.2)	5.6 (19.6)	

IQR, interquartile range; AL, acute leukemia; MDS, myelodysplastic leukemia; Other*, containing lymphoma, chronic myelomonocytic leukemia, granulocytic sarcoma and severe aplastic anemia; CR, complete remission; HCT-CI, Hematopoietic Cell Transplantation-Comorbidity Index; HID, haploidentical; MSD, Matched Sibling Donor; URD, Unrelated Donor; BU, busulfan; TBI, Total-body irradiation.

### Treatment-specific data

Treatment-specific data before anti-CD25 monoclonal antibody administration are detailed in **Supplementary Table 1**. Prior to treatment, The xenopax group had a higher proportion of severe aGVHD, including Grade III (34.4% vs 56.8%) and Grade IV (37.5% vs 10.8%) cases, compared with the control group (p=0.027). Gastrointestinal Stage 4 involvement was also more frequent (34.4% vs 10.8%, p=0.093). sIPTW adjustment successfully balanced baseline characteristics, including aGVHD severity and gastrointestinal Stage 4 involvement, resulting in comparable patient profiles across groups (all p>0.05).

**Table 2** presented detailed information on the anti-CD25 monoclonal antibodies used. The interval between treatment initiation and the onset of aGVHD was shorter in The xenopax cohort (median, 5 versus 9 days, p<0.05). The xenopax group also received a higher median number of administrations (5.5 vs 4.0, p<0.05) and had a greater proportion of patients receiving combined treatment with ruxolitinib (40.6% vs 18.9%, p=0.086). No significant difference was observed between the groups in terms of the combined therapy or the Minnesotar risk score. After sIPTW adjustment, the interval between treatment initiation and aGVHD onset was no longer statistically different (median 7.2 vs 8.17 days, p=0.079), and the median number of administrations was comparable (median 4.0 vs 4.0, p=0.17). The proportion of patients receiving combination therapy with ruxolitinib was also similar between groups (41.1% vs 20.6%, p=0.158).

**Table 2:** Detailed information of anti-CD25 monoclonal antibody.

Characteristics	Before IPTW		P	After IPTW		P
	Basiliximab (n=37)	Xenopax (n=32)		Basiliximab (n=30.1)	Xenopax (n=28.8)	
Combined therapy, n (%)						
no	7 (18.9)	5 (15.6)	0.967	4.2 (13.9)	4.3 (15.0)	0.909
yes	30 (81.1)	27 (84.4)		26.0 (86.1)	24.5 (85.0)	
Ruxolitinib combined, n (%)						
no	30 (81.1)	19 (59.4)	0.086	23.9 (79.4)	17.0 (58.9)	0.158
yes	7 (18.9)	13 (40.6)		6.2 (20.6)	11.8 (41.1)	
aGVHD status before anti-CD25 monoclonal antibody, n (%)						
Grade II	12 (32.4)	9 (28.1)	0.027	10.9 (36.2)	8.9 (30.9)	0.806
Grade III	21 (56.8)	11 (34.4)		14.5 (47.9)	13.1 (45.5)	
Grade IV	4 (10.8)	12 (37.5)		4.8 (15.8)	6.8 (23.6)	
GVHD prophylaxis, n (%)						
CNI MMF MTX	19 (51.4)	22 (68.8)	0.222	13.7 (45.4)	18.6 (64.5)	0.215
CNI MTX	18 (48.6)	10 (31.2)		16.5 (54.6)	10.2 (35.5)	
Minnesotar risk score before treatment, n (%)						
Standard risk	16 (43.2)	11 (34.4)	0.613	12.7 (42.3)	10.2 (35.3)	0.641
High risk	21 (56.8)	21 (65.6)		17.4 (57.7)	18.6 (64.7)	
donor age, n (%)						
≥40	17 (45.9)	16 (50.0)	0.925	14.9 (49.4)	16.6 (57.6)	0.589
<40	20 (54.1)	16 (50.0)		15.3 (50.6)	12.2 (42.4)	
The number of administrations, M(IQR)	4.00 [3.00, 5.00]	5.50 [4.00, 7.25]	<0.001	4.00 [3.00, 5.00]	4.00 [4.00, 6.00]	0.17
Interval between treatment and onset of aGVHD, days (IQR)	9.00 [5.00, 16.00]	5.00 [3.00, 8.25]	0.008	8.17 [4.44, 16.00]	7.02 [3.00, 8.00]	0.079

CNI, calcineurin inhibitor; MMF, mycophenolate mofetil; MTX, methotrexate; MZR, mizoribine; aGVHD, acute graft versus host diseases; MSC, mesenchymal stem cells; CR, complete remission; NR, no remission; PR, partial remission.

Xenopax administration was discontinued for the following reasons: achievement of complete remission (CR) in 21 patients (66%), progression of GVHD in 8 patients (25%), severe infectious complications in 1 patient (3%), cerebral infarction in 1 patient (3%), and loss to follow-up in 1 patient (3%). Similarly, basiliximab treatment ceased due to the achievement of CR in 23 patients (62%), progression of GVHD in 13 patients (35%), and severe infectious complications in 1 patient (3%).

### Efficacy

**Figure 1:**
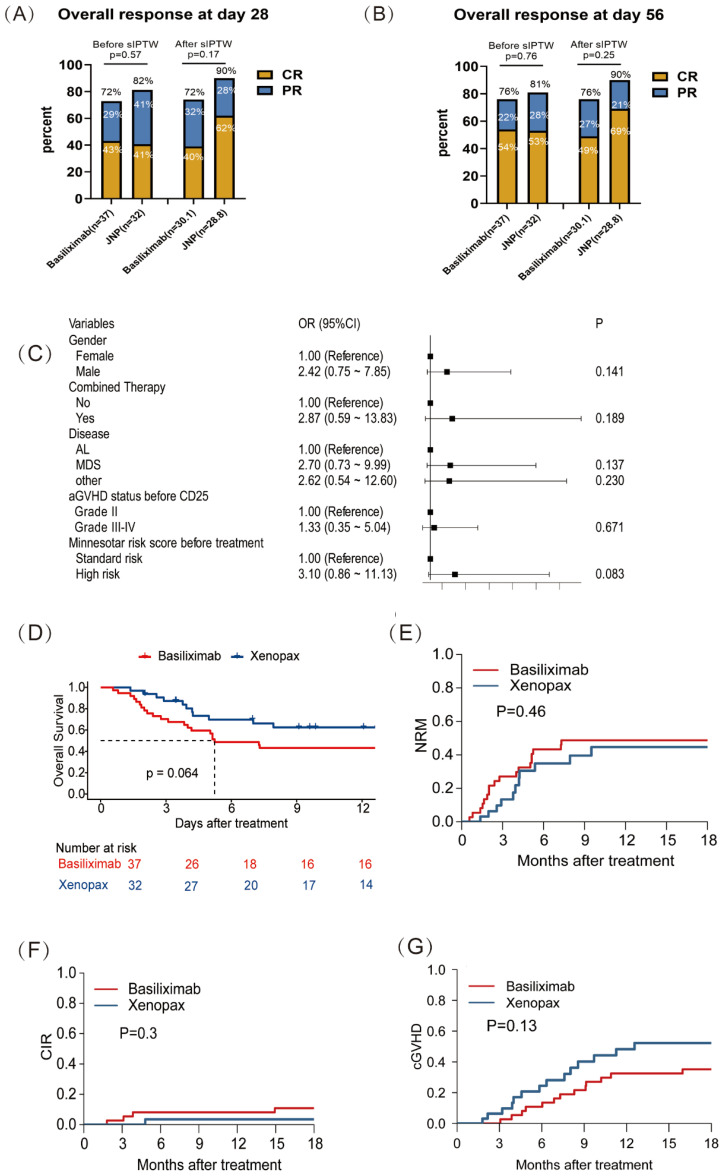
Treatment response and outcome of xenopax and basiliximab in SR-aGVHD patients. **(A)** Overall response rate at day 28 after xenopax and basiliximab treatment; **(B)** Overall response rate at day 56 after xenopax and basiliximab treatment; **(C)** The multivariate logistic regression analysis of the rate of not CR at day 28 after the application of anti-CD25 monoclonal antibody. **(D)** Overall survival rate after xenopax and basiliximab treatment; **(E)** Non-relapse mortality rate after xenopax and basiliximab treatment; **(F)** The cumulative incidence of relapse after xenopax and basiliximab treatment; **(G)** The cumulative incidence of chronic graft versus host disease after xenopax and basiliximab treatment.

On day 28, CR was observed in 13 of 32 patients (41%) in the xenopax group, partial response (PR) in 13 patients (41%), and no response (NR) in 6 patients (18%). In the basiliximab group, CR was achieved in 16 of 37 patients (43%), PR in 11 patients (30%), and NR in 10 patients (27%) (**Figure 1A**). The overall response (OR) rate on day 28 did not differ significantly between the two groups, with rates of 81% (26/32) for xenopax and 73% (27/37) for basiliximab (odds ratio [OR], 1.11; 95% confidence interval [CI], 0.43–2.91; p=0.83). By day 56, CR was achieved in 17 of 32 patients (53%) in the xenopax group, PR in 9 patients (28%), and NR in 6 patients (19%). In the basiliximab group, CR was achieved in 20 of 37 patients (56%), PR in 8 patients (22%), and NR in 9 patients (24%) (**Figure 1B**). On day 56, the OR rates remained similar between the two groups—81% (26/32) for xenopax and 76% (28/37) for basiliximab (OR, 1.04; 95% CI, 0.40–2.68; p=0.94). After adjustment using the augmented sIPTW method, no statistically significant difference in OR rates was observed between the groups at either time point (day 28: OR, 0.40; 95% CI, 0.13–1.30; p=0.13; day 56: OR, 0.43; 95% CI, 0.14–1.40; p=0.16).

Among patients who achieved OR at day 28 (n=53) compared with those who did not (n=16), the proportion receiving any combination therapy was 42 (79%) vs 15 (94%), respectively (p=0.33). The proportion receiving mesenchymal stem cells (MSC) in combination therapy was 35 (66%) vs 14 (88%) (p=0.18), while the proportion receiving ruxolitinib in combination therapy was 14 (26%) vs 6 (38%) (p=0.59). No statistically significant differences were observed across these comparisons.

In the cohort with intestinal involvement (n=60), 29 patients (48%) demonstrated concurrent involvement of additional organs, with 20 of these individuals (69%) achieving an ORR by day 56. Conversely, among the 31 patients (52%) exhibiting isolated intestinal involvement, 26 patients (84%) achieved an ORR by day 56. The difference in ORR by day 56 between patients with isolated intestinal involvement and those with concurrent organ involvement was not statistically significant (p=0.17).

Univariate logistic regression analysis was conducted to evaluate the association of each parameter with the rate of non-CR on day 28 following the administration of the anti-CD25 antibody (**Supplementary Table 2**). The risk factors identified in the univariate analyses were subsequently included in the multivariable logistic regression analysis. The Minnesota score demonstrated the strongest association with the rate of non-CR on day 28; however, this association did not reach conventional statistical significance (p=0.08, **Figure 1C**). After sIPTW adjustment, the univariate analysis suggested that a shorter interval between treatment and the onset of acute GVHD was associated with a higher rate of non-CR on day 28 (p=0.046, **Supplementary Table 2**). Nevertheless, this association was not retained in the multivariable model (p=0.116).

### Safety

Within six months following treatment, infectious complications were observed in 25 patients (78%) in the xenopax cohort and 29 patients (78%) in the basiliximab cohort. There was no significant difference in infection rates between the two cohorts (**Table 3**). Specifically, the incidence rates of CMV viremia, EBV viremia, and other viremias were 53% versus 57% (p=0.95), 3% versus 11% (p=0.45), and 3% versus 8% (p=0.71) in the xenopax and basiliximab cohorts, respectively. Furthermore, the occurrence rates of sepsis, bacterial pneumonia, and other bacterial infections were 6% versus11% (p=0.81), 13% versus 30% (p=0.15), and 6% versus 8% (p=1) in the xenopax and basiliximab cohorts, respectively. The incidence of fungal pneumonia was 6% and 5% (p=1) in the xenopax and basiliximab cohorts, respectively. Neither group exhibited signs of allergy or infusion reactions, and no patients experienced grade 4 or 5 treatment-related adverse events.

**Table 3:** The incidence of infection after xenopax and basiliximab.

Characteristics	Before IPTW		P	After IPTW		P
	Basiliximab (n=37)	Xenopax (n=32)		Basiliximab (n=30.1)	Xenopax (n=28.8)	
Infection, n (%)						
no	8 (21.6)	7 (21.9)	1	4.7 (15.6)	10.2 (35.3)	0.129
yes	29 (78.4)	25 (78.1)		25.5 (84.4)	18.6 (64.7)	
CMV viremia, n (%)						
no	16 (43.2)	15 (46.9)	0.952	14.5 (48.0)	16.7 (57.9)	0.52
yes	21 (56.8)	17 (53.1)		15.7 (52.0)	12.1 (42.1)	
CMV disease, n (%)						
no	36 (97.3)	31 (96.9)	1	29.0 (96.3)	28.3 (98.3)	0.585
yes	1 (2.7)	1 (3.1)		1.1 (3.7)	0.5 (1.7)	
EBV viremia, n (%)						
no	33 (89.2)	31 (96.9)	0.446	26.9 (89.2)	28.3 (98.3)	0.068
yes	4 (10.8)	1 (3.1)		3.2 (10.8)	0.5 (1.7)	
Other viremia, n (%)						
no	34 (91.9)	31 (96.9)	0.714	26.4 (87.5)	28.3 (98.4)	0.046
yes	3 (8.1)	1 (3.1)		3.8 (12.5)	0.5 (1.6)	
Sepsis, n (%)						
no	33 (89.2)	30 (93.8)	0.809	27.4 (91.0)	27.8 (96.7)	0.249
yes	4 (10.8)	2 (6.2)		2.7 (9.0)	1.0 (3.3)	
Pneumonia, n (%)						
no	26 (70.3)	28 (87.5)	0.151	18.6 (61.9)	26.9 (93.4)	0.001
yes	11 (29.7)	4 (12.5)		11.5 (38.1)	1.9 (6.6)	
Other infection, n (%)						
no	34 (91.9)	30 (93.8)	1	28.2 (93.6)	26.3 (91.4)	0.766
yes	3 (8.1)	2 (6.2)		1.9 (6.4)	2.5 (8.6)	
Fungal pneumonia, n (%)						
no	35 (94.6)	30 (93.8)	1	28.8 (95.4)	26.8 (93.2)	0.706
yes	2 (5.4)	2 (6.2)		1.4 (4.6)	2.0 (6.8)	

After applying sIPTW, however, the analysis revealed that the xenopax group had a lower incidence of pneumonia (p=0.001) and other viremia (p=0.046) than the basiliximab group, though no significant differences were found for the remaining infectious outcomes (**Table 3**).

### Follow-up and outcomes

The median follow-up duration for the study population was 7.3 months (range, 0.6–104 months). In the unadjusted analysis, the 1-year OS rate was 63% (95% CI, 51–86%) in the xenopax cohort compared with 43% (95% CI, 30–63%) in the basiliximab cohort (p=0.06; **Figure 1D**). Following adjustment using sIPTW, the 1-year OS rate was 64% (95% CI, 43–96%) for xenopax versus 40% (95% CI, 25–63%) for basiliximab (p=0.114, **Supplementary Figure 1A**).

There were no significant differences in NRM or CIR between the xenopax and basiliximab cohorts. For 1-year NRM, the rates were 45% (95% CI: 24–64%) versus 48% (95% CI: 32–64%) (p=0.46, **Figure 1E**) in the xenopax and basiliximab cohorts, respectively. For the 1-year CIR, the rates were 3% (95% CI: 0–16%) versus 5% (95% CI: 2–20%) (p=0.30, **Figure 1F**) in The xenopax and basiliximab cohorts, respectively. The incidence of cGVHD did not significantly differ between The xenopax and basiliximab cohorts, with 1-year rates of 48% (95% CI: 28–66%) and 32% (95% CI: 18–48%), respectively (p=0.13; **Figure 1G**). After adjustment using sIPTW, no statistically significant differences were observed between the xenopax and basiliximab cohorts in 1-year NRM (p=0.23; **Supplementary Figure 1B**), CIR (p=0.09; **Supplementary Figure 1C**), or cGVHD (p=0.47; **Supplementary Figure 1D**).

Moreover, the treatment responses to anti-CD25 monoclonal antibodies on days 28 and 56 significantly affected OS and NRM (**Supplementary Figures 1E–H**).

## Discussion

SR-aGVHD is a fatal and refractory complication that can occur after allo-HSCT (15), and currently, there is no established standard for second-line therapies for SR-aGVHD (2, 16). Basiliximab is known to specifically target the IL-2Rα receptor, thereby inhibiting T lymphocyte activation with high affinity (17). Basiliximab is the most widely used CD25 monoclonal antibody and exhibits a high response rate among SR-aGVHD patients (7). Although the immunogenicity of therapeutic antibodies is a general consideration in their clinical application, the development of various antibody constructs, including chimeric and humanized versions, warrants direct comparative evaluation. Furthermore, factors such as the limited availability of basiliximab in certain regions create a need for effective and accessible alternatives. Therefore, this study was designed to compare the clinical efficacy and safety profile of xenopax with basiliximab for GVHD treatment.

Due to the limitations inherent in the historical cohort design and the small sample size, baseline characteristics between the xenopax and basiliximab groups were not well balanced. Prior to treatment, the xenopax group exhibited a higher proportion of severe aGVHD, including Grade III (34.4% vs. 56.8%) and Grade IV (37.5% vs. 10.8%) cases, compared with the basiliximab group (p=0.027). Furthermore, xenopax-treated patients demonstrated greater utilization of ruxolitinib combination therapy (p=0.086), shorter intervals from aGVHD onset to treatment initiation (p=0.045), and receipt of more doses (p=0.002). To mitigate this selection bias, we applied a sIPTW method based on propensity scores. All study outcomes were subsequently re-evaluated following this statistical adjustment.

Our analysis demonstrated a comparable efficacy and safety profile for xenopax relative to basiliximab. The OR rates were similar between the groups at day 28 (81% vs. 73%; p=0.83) and day 56 (81% vs. 76%; p=0.94). While not statistically significant, 1-year OS was 63% in the xenopax arm versus 43% in the control arm (p=0.06). Similarly, 1-year NRM rates did not differ significantly (45% vs. 48%; p=0.46). Following sIPTW adjustment to account for baseline imbalances, these outcomes remained comparable, with no significant differences observed.

Within six months post-treatment, infectious complications were reported in 25 (78.1%) and 29 (78.4%) patients in the xenopax and basiliximab cohorts, respectively. The overall incidence of bacterial, fungal, or viral infections did not differ significantly between the groups. Notably, after sIPTW adjustment, the xenopax group exhibited a significantly lower incidence of both pneumonia (p=0.001) and other viremia (p=0.046) than the basiliximab group. However, this observation should be interpreted with caution, as its robustness is limited by both the small number of observed events and the potential for unmeasured bias from the sIPTW adjustment. Therefore, these findings necessitate confirmation through larger, prospective follow-up studies.

Previous research has identified liver involvement as an independent risk factor for poor treatment response on day 28 in patients with SR-aGVHD receiving xenotherapy (10). In our cohort, a high Minnesota risk score was significantly associated with failure to achieve CR on day 28 in patients treated with anti-CD25 monoclonal antibodies in the univariate logistic regression analysis (p=0.01), however, this association did not reach statistical significance in the multivariate model (p=0.083). These findings indicate that while the Minnesota risk score may have potential prognostic value for predicting treatment outcomes with anti-CD25 monoclonal antibodies, further studies with larger sample sizes are warranted to confirm its independent predictive capability.

In 2024, the European Society for Blood and Marrow Transplantation (EBMT) recommended ruxolitinib as the primary treatment for SR-aGVHD, establishing it as a new standard of care (1). The efficacy of ruxolitinib combined with an anti-CD25 monoclonal antibody (xenopax, n=13; basiliximab, n=7) was evaluated in a cohort of 20 patients. The ORR on day 28 was 70%, comprising a CR rate of 35% (n=7) and a PR rate of 35% (n=7).

This study had several limitations. First, the small sample size and reliance on historical cohorts resulted in unmatched groups with respect to baseline characteristics, introducing the potential for confounding bias. Second, the research was conducted at a single center, necessitating the use of multicenter data for further validation. Third, immunogenicity was not assessed in our study, and our clinical data did not demonstrate any safety advantage of xenopax over basiliximab with respect to allergic reactions. Finally, the majority of participants were male, individuals with low hematopoietic cell transplantation-comorbidity index (HCT-CI) scores, and patients who had received transplants from haploidentical donors; therefore, it may be difficult to generalize the findings to other populations due to this limitation.

In conclusion, this study represents the first comparative analysis of the efficacy and safety of xenopax and basiliximab. These findings support the use of xenopax in the treatment of SR-aGVHD, increase the understanding of its clinical application, and indicate that the Minnesota risk score may serve as a predictor of the efficacy of anti-CD25 monoclonal antibodies. Further multicenter prospective studies are warranted to compare xenopax with alternative therapeutic options.

## Data Availability

The raw data supporting the conclusions of this article will be made available by the authors, without undue reservation.

## Supplementary material

Supplementary Table 1 The aGVHD status before the application of anti-CD25 Monoclonal Antibody.

Supplementary Table 2 The Univariate logistic regression analysis of the rate of non-CR at day 28 after the application of anti-CD25 monoclonal antibody.

Supplementary Figure 1 Outcomes of xenopax and basiliximab after adjustment using sIPTW. **(A)** Overall survival rate after xenopax and basiliximab treatment; **(B)** Non-relapse mortality rate after xenopax and basiliximab treatment; **(C)** The cumulative incidence of relapse after xenopax and basiliximab treatment; **(D)** The cumulative incidence of chronic graft versus host disease after xenopax and basiliximab treatment. The outcome of SR-aGVHD patients grouped by the treatment response of anti-CD25 monoclonal antibody. **(E)** OS grouped by the treatment response at day 28; **(F)** OS grouped by the treatment response at day 56; (G) NRM grouped by the treatment response at day 28; **(H)** NRM grouped by the treatment response at day 56.
