# Supplementary material for: Comparison of chimeric mouse-human and humanized anti-CD25 monoclonal antibodies for steroid-refractory acute graft-versus-host disease
Source: Front Immunol. 2026 Jan 2;16:1660452. doi: 10.3389/fimmu.2025.1660452 (PMC12808378; doi:10.3389/fimmu.2025.1660452)
Supplement: Supplementary Table 1 — The aGVHD status before the application of anti-CD25 Monoclonal Antibody. [file DataSheet1.docx]

Supplementary table1. The aGVHD status before the application of anti-CD25 Monoclonal Antibody.

|  | Before IPTW | |  | After IPTW | |  |
| --- | --- | --- | --- | --- | --- | --- |
| Characteristics | Basiliximab  (n=37) | Xenopax  (n=32) | p | Basiliximab  (n=30.1) | Xenopax  (n=28.8) | p |
| aGVHD status before CD25, n (%) | | | |  |  |  |
| Grade II | 12 (32.4) | 9 (28.1) | 0.027 | 10.9 (36.2) | 8.9 (30.9) | 0.806 |
| Grade III | 21 (56.8) | 11 (34.4) | | 14.5 (47.9) | 13.1 (45.5) | |
| Grade IV | 4 (10.8) | 12 (37.5) | | 4.8 (15.8) | 6.8 (23.6) | |
| Minnesota risk score before CD25, n (%) | | | |  |  |  |
| 1 | 16 (43.2) | 11 (34.4) | 0.613 | 12.7 (42.3) | 10.2 (35.3) | 0.641 |
| 2 | 21 (56.8) | 21 (65.6) | | 17.4 (57.7) | 18.6 (64.7) | |
| Skin involvement before CD25, n (%) | | | |  |  |  |
| Stage 0 | 20 (54.1) | 21 (65.6) | 0.352 | 14.2 (47.1) | 21.6 (75.0) | 0.074 |
| Stage 1 | 8 (21.6) | 4 (12.5) | | 7.4 (24.5) | 2.4 ( 8.2) | |
| Stage 2 | 6 (16.2) | 4 (12.5) | | 6.8 (22.5) | 1.9 ( 6.6) | |
| Stage 3 | 3 ( 8.1) | 1 ( 3.1) |  | 1.7 ( 5.8) | 2.0 ( 6.9) | |
| Stage 4 | 0 ( 0.0) | 2 ( 6.2) |  | 0.0 ( 0.0) | 0.9 ( 3.3) | |
| Gastrointestinal involvement before CD25, n (%) | | | | |  |  |
| Stage 0 | 5 (13.5) | 4 (12.5) | 0.093 | 3.0 (10.1) | 7.7 (26.9) | 0.491 |
| Stage 1 | 9 (24.3) | 9 (28.1) | | 9.1 (30.1) | 7.4 (25.6) | |
| Stage 2 | 10 (27.0) | 3 ( 9.4) |  | 7.3 (24.3) | 3.4 (11.9) | |
| Stage 3 | 9 (24.3) | 5 (15.6) | | 6.0 (19.8) | 3.9 (13.6) | |
| Stage 4 | 4 (10.8) | 11 (34.4) | | 4.8 (15.8) | 6.3 (21.9) | |
| liver involvement before CD25, n (%) | | | |  |  |  |
| Stage 0 | 25 (67.6) | 26 (81.2) | 0.378 | 21.5 (71.3) | 20.0 (69.4) | 0.885 |
| Stage 1 | 4 (10.8) | 2 ( 6.2) |  | 2.8 ( 9.4) | 2.6 ( 9.0) | |
| Stage 2 | 5 (13.5) | 1 ( 3.1) |  | 3.9 (13.0) | 4.8 (16.7) | |
| Stage 3 | 3 ( 8.1) | 2 ( 6.2) |  | 1.9 ( 6.4) | 0.9 ( 3.3) | |
| Stage 4 | 0 ( 0.0) | 1 ( 3.1) |  | 0.0 ( 0.0) | 0.5 ( 1.6) | |
| No. of aGVHD involved organs before CD25 , n (%) | | | | |  |  |
| 1 | 17 (45.9) | 21 (65.6) | 0.196 | 11.4 (37.8) | 21.5 (74.6) | 0.011 |
| 2 | 16 (43.2) | 10 (31.2) | | 16.0 (52.9) | 6.9 (23.8) | |
| 3 | 4 (10.8) | 1 ( 3.1) |  | 2.8 ( 9.3) | 0.5 ( 1.6) | |

Supplementary table2. The Univariate logistic regression analysis of the rate of non-CR at day 28 after the application of anti-CD25 monoclonal antibody.

|  | Before IPTW | |  | After IPTW | |
| --- | --- | --- | --- | --- | --- |
| Variables | P | OR (95%CI) |  | P | OR (95%CI) |
| Age of patients |  |  |  |  | |
| ≥40 |  | 1.00 (Reference) |  |  | 1.00 (Reference) |
| ＜40 | 0.49 | 0.71(0.27~1.87) |  | 0.23 | 0.49(0.15~1.57) |
| Age of donors |  |  |  |  |  |
| ≥40 |  | 1.00 (Reference) |  |  | 1.00 (Reference) |
| ＜40 | 0.67 | 0.81(0.31~2.12) |  | 0.78 | 1.19(0.36~3.93) |
| Gender |  |  |  |  |  |
| Female |  | 1.00 (Reference) |  |  | 1.00 (Reference) |
| Male | 0.06 | 2.82(0.97~8.24) |  | 0.02 | 4.67(1.30~16.85) |
| Group |  |  |  |  |  |
| Basiliximab |  | 1.00 (Reference) |  |  | 1.00 (Reference) |
| Xenopax | 0.83 | 1.11(0.43~2.91) |  | 0.13 | 0.40(0.13~1.30) |
| Disease |  |  |  |  |  |
| AL |  | 1.00 (Reference) |  |  | 1.00 (Reference) |
| MDS | 0.07 | 3.00(0.90~10.01) |  | 0.04 | 4.48(1.07~18.70) |
| other | 0.81 | 1.17(0.33~4.16) |  | 0.92 | 1.08(0.25~4.73) |
| Status before transplantation |  |  |  |  |  |
| CR1 |  | 1.00 (Reference) |  |  | 1.00 (Reference) |
| No chemotherapy | 0.47 | 1.67(0.42~6.64) |  | 0.30 | 2.34(0.48~11.50) |
| Not CR1 | 0.78 | 1.17(0.40~3.38) |  | 0.75 | 0.80(0.21~3.10) |
| HCT-CI |  |  |  |  |  |
| 0 |  | 1.00 (Reference) |  |  | 1.00 (Reference) |
| ≥1 | 0.24 | 1.89(0.65~5.47) |  | 0.87 | 0.89(0.23~3.43) |
| HLA |  |  |  |  |  |
| Haploidentical |  | 1.00 (Reference) |  |  | 1.00 (Reference) |
| HLA-matched | 0.77 | 1.16(0.44~3.05) |  | 0.96 | 1.03(0.31~3.46) |
| Sex match |  |  |  |  |  |
| Female-male |  | 1.00 (Reference) |  |  | 1.00 (Reference) |
| Not Female-male | 0.87 | 1.10(0.35~3.39) |  | 0.43 | 1.70(0.46~6.36) |
| Conditioning regimen |  |  |  |  |  |
| BU-based |  | 1.00 (Reference) |  |  | 1.00 (Reference) |
| TBI-based | 0.36 | 0.55(0.15~2.01) |  | 0.36 | 0.49(0.11~2.22) |
| MNCs |  |  |  |  |  |
| ＜9.95 |  | 1.00 (Reference) |  |  | 1.00 (Reference) |
| ≥9.95 | 0.89 | 0.93(0.36~2.43) |  | 0.49 | 0.66(0.20~2.18) |
| CD34 |  |  |  |  |  |
| ＜3..28 |  | 1.00 (Reference) |  |  | 1.00 (Reference) |
| ≥3.28 | 0.27 | 0.58(0.22~1.52) |  | 0.21 | 0.46(0.14~1.52) |
| aGVHD status before CD25 |  |  |  |  |  |
| Grade 2 |  | 1.00 (Reference) |  |  | 1.00 (Reference) |
| Grade 3-4 | 0.10 | 2.43(0.85~6.93) |  | 0.56 | 1.49(0.40~5.61) |
| Interval between treatment and onset of aGVHD |  |  |  |  |  |
| ≥7 |  | 1.00 (Reference) |  |  | 1.00 (Reference) |
| ＜7 | 0.14 | 2.10(0.78~5.63) |  | 0.05 | 3.38(1.05~10.91) |
| Dose of methylprednisolone |  |  |  |  |  |
| ≥1mg/kg |  | 1.00 (Reference) |  |  | 1.00 (Reference) |
| < 1mg/kg | 0.24 | 1.82(0.67~4.96) |  | 0.95 | 0.96(0.27~3.35) |
| Minnesota risk score before CD25 |  |  |  |  |  |
| Standard risk |  | 1.00 (Reference) |  |  | 1.00 (Reference) |
| High risk | 0.01 | 4.25(1.52~11.89) |  | 0.17 | 2.58(0.67~9.91) |
| The number of administrations |  |  |  |  |  |
| ≥4 |  | 1.00 (Reference) |  |  | 1.00 (Reference) |
| ＜4 | 0.17 | 0.48(0.17~1.37) |  | 0.77 | 0.84(0.26~2.70) |
| Combined therapy |  |  |  |  |  |
| no |  | 1.00 (Reference) |  |  | 1.00 (Reference) |
| yes | 0.07 | 3.43(0.92~12.78) |  | 0.09 | 3.60(0.84~15.44) |
| Ruxolitinib Combined |  |  |  |  |  |
| no |  | 1.00 (Reference) |  |  | 1.00 (Reference) |
| yes | 0.45 | 1.51(0.52~4.45) |  | 0.93 | 0.94(0.22~3.94) |
| Active infection before treatment |  |  |  |  |  |
| no |  | 1.00 (Reference) |  |  | 1.00 (Reference) |
| yes | 0.59 | 0.76(0.29~2.04) |  | 0.94 | 0.95(0.29~3.18) |

Abbreviations: AL, acute leukemia; MDS, myelodysplastic leukemia; CR, complete remission; Other, containing lymphoma, chronic myeloid leukemia, and severe aplastic anemia; HCT-CI, Hematopoietic Cell Transplantation-Comorbidity Index; HID, haploidentical; MSD, Matched Sibling Donor; URD, Unrelated Donor; BU, busulfan; TBI, Total-body irradiation; MNCs, mononuclear cells; aGVHD, acute graft versus host diseases;


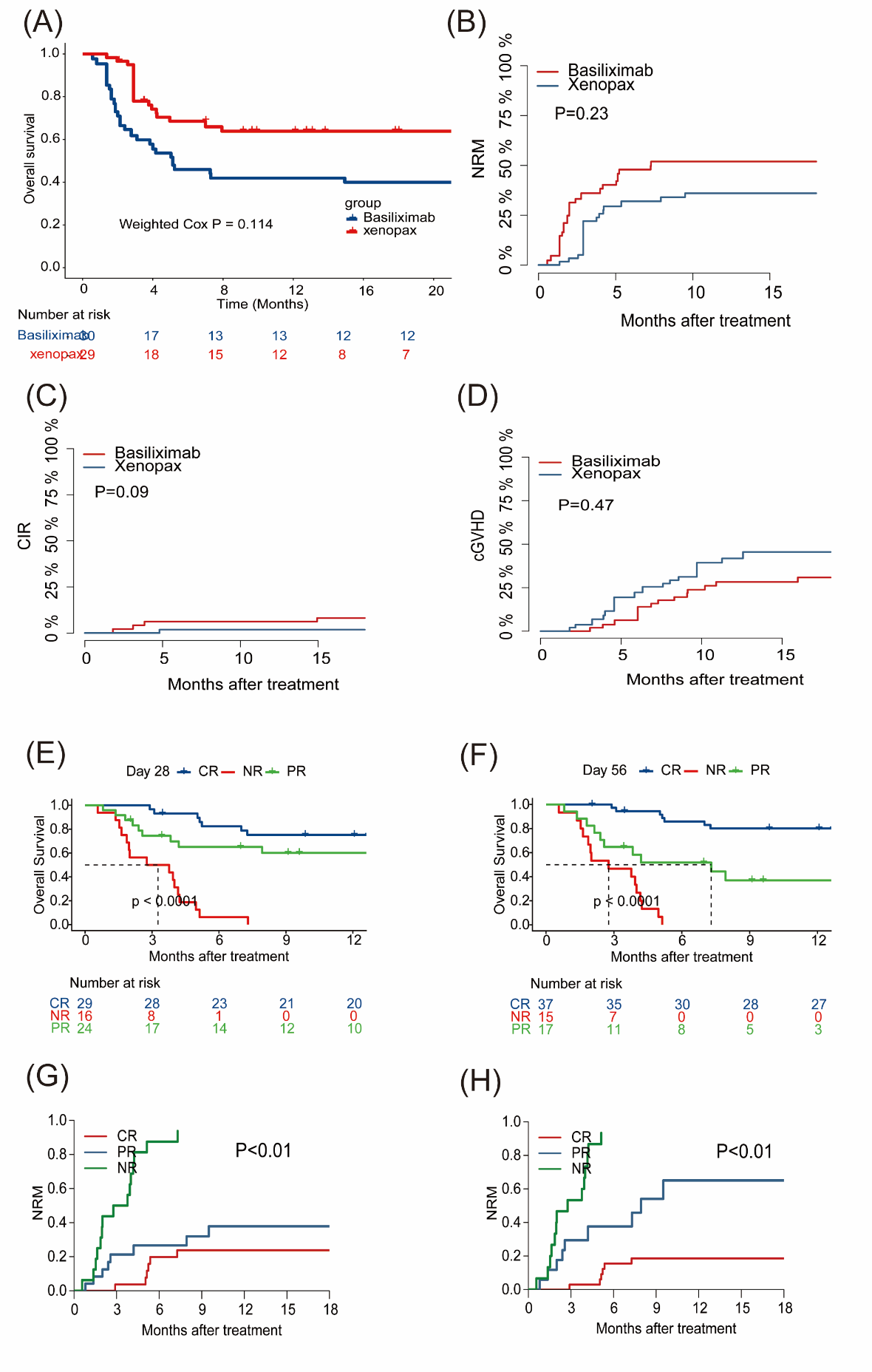


Supplementary figure 1. Outcomes of xenopax and basiliximab after adjustment using sIPTW.

(A) Overall survival rate after xenopax and basiliximab treatment; (B) Non-relapse mortality rate after xenopax and basiliximab treatment; (C) The cumulative incidence of relapse after xenopax and basiliximab treatment; (D) The cumulative incidence of chronic graft versus host disease after xenopax and basiliximab treatment;

The outcome of SR-aGVHD patients grouped by the treatment response of anti-CD25 monoclonal antibody. (E) OS grouped by the treatment response at day 28; (F) OS grouped by the treatment response at day 56; (G) NRM grouped by the treatment response at day 28; (H) NRM grouped by the treatment response at day 56.
